# Struck by lightning or slowly suffocating – gendered trajectories into depression

**DOI:** 10.1186/1471-2296-10-56

**Published:** 2009-08-11

**Authors:** Ulla Danielsson, Carita Bengs, Arja Lehti, Anne Hammarström, Eva E Johansson

**Affiliations:** 1 Department of Public Health and Clinical Medicine, Division of Family Medicine, Umeå University, Umeå, Sweden; 2 Department of Sociology, Umeå University, Umeå, Sweden

## Abstract

**Background:**

In family practice depression is a common mental health problem and one with marked gender differences; women are diagnosed as depressed twice as often as men. A more comprehensive explanatory model of depression that can give an understanding of, and tools for changing, this gender difference is called for. This study explores how primary care patients experience, understand and explain their depression.

**Methods:**

Twenty men and women of varying ages and socioeconomic backgrounds diagnosed with depression according to ICD-10 were interviewed in-depth. Data were assessed and analyzed using Grounded Theory.

**Results:**

The core category that emerged from analysis was "Gendered trajectories into depression". Thereto, four categories were identified – "Struck by lightning", "Nagging darkness", "Blackout" and "Slowly suffocating" – and presented as symbolic illness narratives that showed gendered patterns. Most of the men in our study considered that their bodies were suddenly "struck" by external circumstances beyond their control. The stories of study women were more diverse, reflecting all four illness narratives. However, the dominant pattern was that women thought that their depression emanated from internal factors, from their own personality or ways of handling life. The women were more preoccupied with shame and guilt, and conveyed a greater sense of personal responsibility and concern with relationships.

**Conclusion:**

Recognizing gendered narratives of illness in clinical consultation may have a salutary potential, making more visible depression among men while relieving self-blame among women, and thereby encouraging the development of healthier practices of how to be a man or a woman.

## Background

According to Swedish national statistics, one in two women and one in four men are considered to be depressed at some point in their lives [1]. Gender differences in mental health have long been noted. Thirty years ago, Weissman and Klerman showed that women were diagnosed as depressed twice as often as men [2]. Medical discourse focuses on biomedical explanations of such gender differences, but according to many reviews socio-cultural and psychological factors also influence depression [3,4]. A number of studies call for comprehensive explanatory models that can give an understanding of, and tools for changing, this gender difference in depression [4-6].

Considerable research has been conducted on women and depression. Women's own experiences and voices have been in focus [7,8]. Less is known about men's experiences of depression. Recently a number of studies have focused on men [9-13] and also on both men and women [14-20]. It has been suggested that depressive symptoms in men are often undiagnosed and untreated [21].

In our effort to explore men's and women's own explanations of depression, we draw on a framework of gender theory and social constructionism. According to gender theory, being a man or a woman is a condition actively under construction and formed in relation to other people. Gender is therefore historically and culturally variable and thus open for change [22]. In daily relationships, gender is continually negotiated and renegotiated in the process of "doing gender" in which we react to cultural norms of how to be a man or a woman [23]. Men and women are active agents in the process of thinking, acting and performing gender, making possible divergent patterns of being a man or a woman [22]. Gender actions can be studied in different contexts including health [24,25].

Connell proposes a relational model in which gender denotes not only sets of relations between men and women, but also relations between men and between women. Power asymmetry is central to each of these sets of relationships [22]. Hegemonic masculinity, the culturally idealized form of masculine character, can be understood as patterns and practices that support men's dominance over women and the subordination of certain groups of men [26]. Between men and women such power asymmetry comes to light when men are treated as the norm, creating a risk that women be viewed as deviant. With regard to mental disorders for example, Busfield [27] has explored how women are seen as problematic. Between men, the patterning of contemporary masculinities may also have health consequences. For instance, dominating masculinity norms may disparage open expressions of vulnerability and sadness [13]. In so doing, such norms may well encourage a masking of depressive symptoms and play a role in men's suicidal behavior [9,21,28].

Social constructionism regards all knowledge, including biomedical, as socially constructed. It does not attempt to present a single, unified account about disease and the body. Rather, social constructionism furthers an eclectic approach, acknowledging as valid, knowledge based on experience as well as scientific evidence [29]. Health and illness can thus be understood on the basis of expert knowledge as well as lay beliefs. A highly medicalized and biological understanding of depression may not always coincide with the everyday experience of the patient [30].

Still, bio-medical explanations have been found to help women manage the stigma of depression [7]. Kangas [16] demonstrated how people suffering from depression cope with expert knowledge and personal understanding by constructing storylines and explanations that give their illness meaning. The result of mutually exchanged information may contribute to evolving and changing explanatory models. Kleinman [31] emphasizes the joint venture of negotiation between doctors and patients in which patients express their thoughts and doctors openly examine the explanations, until a mutual understanding about cause and treatment arises. This approach emphasizes the central role of the dialogue itself. Successful negotiations may lead to a mutual understanding that enhances the prospect of recovery. Similarly, Malterud and Hollnagel [32] present an awareness model that avoids humiliation by jointly exploring the doctor's and the patient's agendas and balancing the emotions and rationality of both. Pollock [17] also underscores the consultation as a social encounter, calling attention to the importance of "maintaining face" in the presentation of depression, which may otherwise constrain both detection and treatment.

Using the above framework, this study explores how primary care patients experience, understand and explain their depression. In particular, this study addresses the question, "What is the impact of gender in this process?"

## Methods

### Recruitment of informants and data analysis

Informants were recruited in a primary care setting. We wanted to interview women and men who had been considered depressed in a medical context and at the same time listen to their personal voices and narratives of experience. Recruitment took place during a two-year period (2002–2004) among patients at two health care centers in a university town in northern Sweden. All informants had been diagnosed with depression according to ICD-10 criteria [33]. Interviews were conducted six months after the beginning of treatment (anti-depressants and/or psychotherapy), in that we wanted the experience of depression to be fresh in mind but not acute.

The patients were informed about how data were to be collected and reported, and that confidentiality would be respected. Both oral and written informed consent was obtained. The study was approved by the Research Ethics Committee at Umeå University (Dnr: 00 – 312).

Informants were strategically selected by the researchers (with the help of physician colleagues) so as to obtain narratives of experience from varying social contexts. Initially any patient who fitted the inclusion criteria was included. As the study progressed, however, we actively selected informants to mirror both genders, as well as varying ages, occupations and marital status. Recruitment was also guided by theoretical sampling – data collection and analysis were performed simultaneously and we used emerging themes to choose new informants. For instance, initially more women than men were recruited. However, as certain gendered response patterns appeared in the analysis, we invited additional men so as to explore more thoroughly gender similarities, variations and exceptions.

The number of participants was not decided upon in advance. Recruitment continued until no further themes concerning gender similarities or differences were identified and we thereby considered our material to be saturated. At that point, we had interviewed ten women and ten men. Men aged 24–66 years; women, 19–54 years. Half of the men were academics or white-collar workers, such as entrepreneurs or consultants. Likewise half of the women were academics. The remaining half of both men and women were blue-collar workers, such as home help assistants or janitors. Five of the women were single; five were married/cohabiting, of whom four had children. All of the men had been married/cohabiting, but two were divorced/separated at the time of interview. Eight of the men had children, including the two who were separated. With one exception all of the informants were born in Sweden.

Interviews were conducted by either UD or EJ on a single occasion at the health care center or in the informant's home. The interviews were semi-structured and designed to cover the domains of Malterud's key-questions [34]: Why did you initially seek health care? What was your understanding of the reasons for falling ill? What were your expectations and experiences of treatment? By posing open questions and by following up with: How do you mean? Can you explain? or Can you give an example? we encouraged the informants to narrate their specific personal experiences in their own words. Each interview lasted 1–2 hours, was audio taped and transcribed verbatim.

Data collection and analysis were performed according to Grounded Theory guidelines [35,36]. In addition, we were inspired by Clarke's situational mapping [37] and by Ragin's [38] strategy of applying theory to data. First, each interview was read and openly coded independently by UD and EJ, based on transcripts in the original Swedish. Thereafter, the two interviewers met, re-examined and compared codes, and devised preliminary categories. Tentative questions were posed to the data; e.g., What might the understanding behind this expression be? How is the story told? How is gender negotiated? In this way, ideas from the ongoing process of analysis were brought to the next interview and further revised and developed.

Toward the end of the interview process, the entire research team examined the transcripts once again. Codes and preliminary categories were scrutinized and compared until consensus was reached. During the entire process, we took active steps to maintain the scientific rigor and trustworthiness of our qualitative data [39]. Dependability was secured by doing interviews and analysis simultaneously in an emerging design. Confirmability was assured by searching actively for negative cases and exceptions, and by openly displaying direct quotations (translated below into English).

A core category and four categories central to the data were identified. These are presented as four symbolic illness narratives. In the presentation of findings, codes and categories are shown in italics the first time they appear.

## Results

When informants were asked, "Why do you think you became depressed?" most of them began by saying that they could not see any specific reason. Moreover, at first they had not even realized they were suffering from depression. Rather, it was an uncertainty and vague sense of discomfort without a clear cause that was the problem. Many informants used the same expression – "I can't put my finger on it. That's the problem". However, as the interview progressed the informants recounted a variety of incidents and personal circumstances that may have contributed to their depression; such as traumatic life events, family background, seasonal influences (darkness or light), work-related pressures, bullying, physical experiences, feelings, and specific personality traits.

**Figure 1:**
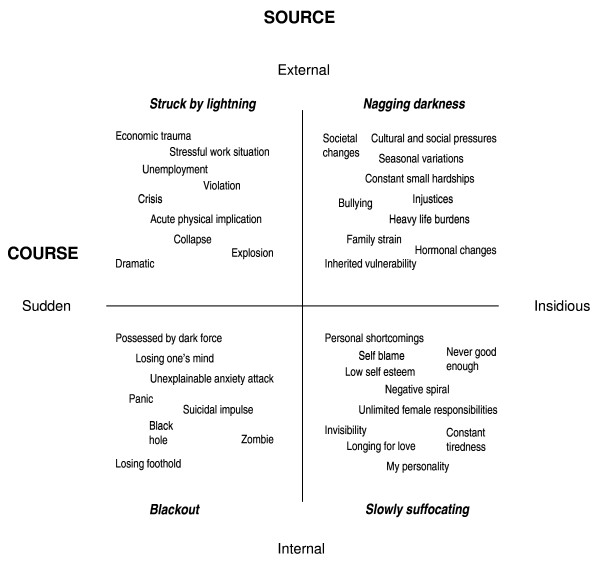
**Gendered trajectories into depression – four symbolic illness narratives with a taxonomy of the experienced source and course of depression as expressed by the informants**.

In our analysis, we explored similarities and differences in the informants' narratives. The core category, *gendered trajectories into depression*, describes men's and women's varying experiences through four symbolic narratives. The properties, *course *and *source*, and their dimensions constitute the frames of our model (Figure 1).

### Course and source of depression

Codes, such as *dramatically, traumatically, successively *or *slowly darker*, from the open coding of the interviews illustrated how the illness occurred and described the *course *of the illness. Time was a central dimension of course; the onset was described along an axis from *sudden *to *insidious*.

The informants' ideas about what might have contributed to their distress and where it came from hinted at the *source *of the illness. Sources included *stressful work-situations, heavy life burdens*, *inner demons*, and *my personality*. The source had an *external – internal *dimension; i.e., was attributed either to an outside force affecting the body or to a mental force within.

In the four-field model (Figure 1), the two properties (course and source) and their dimensions (external-internal and sudden-insidious) form the axes of the model. The four fields display the subcategories of how the informants explained their illness as well as their mode of falling ill. Four symbolic illness narratives, named by the researchers, represent the categories: *Struck by lightning *(external source, sudden course), *Nagging darkness *(external source, insidious course), *Blackout *(internal source, sudden course), and *Slowly suffocating *(internal source, insidious course).

Table 1 shows an example of the coding process, presenting a text excerpt followed by open codes, subcategories, and final resolution into "Struck by lightning", one of the four categories.

**Table 1:** Illustration of the emergence of the category "Struck by lightning"

Text	Open code	Sub-category	Category
"I simply collapsed. It was at night and I was going to the toilet.	Collapse At night	Collapse	Struck by lightning
I jumped out of bed, passed out, fell and hit the bedpost and punctured a lung and broke a few ribs.	Jumped out of bed Passed out Fell Hit Punctured a lung Broke a few ribs	Acute Physical trauma	
As I did in those days, I lay at the intensive ward on Friday, and Monday I was back at work."	Intensive care Back at work	Stressful work-situation	

Although the informants' narratives displayed variations, gendered trajectories into depression were still revealed. In the following, the different symbolic illness narratives are further elaborated and illustrated by quotations from the interviews.

### Struck by lightning

The narratives that characterized the depression as "Struck by lightning" were told mostly by men. In all these stories the course was described as "sudden" and the source as "external". The informants were affected by circumstances beyond their control. Examples included employer bankruptcy, stock market loss, accusations of sexual harassment or, most commonly, insufferable assignments at work. One man described a combination of these circumstances when he suddenly found himself unemployed and with huge debts:

*I worked for [X-constructors] and they went bankrupt. we, the employees had bought convertibles. so I lost about 56 000 at one go. I had bought a house at the same time and they told us that because of the bankruptcy we would be unemployed*.

Another man, father of two children and entrepreneur, talked about how he drove himself so hard that he almost risked his life:

*I drove double races toward the end... I looked in my calendar and it's totally horrendous how I ever thought I could manage...but I was out of it... I jumped the red lights*.

The informants often recalled the exact moment and situation when they "fell ill" as an unexpected and frightening experience, like a bolt from the blue, often with acute physical implications. They presented physical symptoms, such as chest pain, stomach problems, and breathing difficulties, often described in dramatic terms. One woman compared falling ill to an explosion:

*I had a physical pain in my stomach. It felt like a hole, as if something had exploded, there is nothing left. I am empty*.

A man recounted the moment as a collapse:

*I simply collapsed. It was at night and I was going to the toilet. I jumped out of bed, passed out, fell and hit the bedpost and punctured a lung and broke a few ribs. As I did in those days, I lay at the intensive ward on Friday, and Monday I was back at work*.

All informants in this category described themselves as hardworking and found it difficult to set limits. "I could've worked myself to death" was a recurrent statement. Men in this category gave priority to their work and experienced intense work-related pressure and stress. Family issues were rarely mentioned as the source of illness, although all but one man were parents and had children at home. Leisure time and family responsibilities were seen instead as disturbing hindrances to work, as one man stated:

Weekends, God, how boring. Friday was nice, but Saturday mornings I woke up and, gosh, just stayed at home for the sake of being at home in case the children needed food or...

He described further how he was unable to take pleasure in his children. He felt he was supervising them rather than caring for them.

Narratives of the two women in this category, both with careers in the academic field, touched on dual spheres – home and work. Still, it was work-related pressures that were the source of distress. One of the women saw a clear correlation between her ill-health and her workplace that suddenly had become dysfunctional with "a bullying boss and fellow workers". The illness manifested itself as totally physical:

*I couldn't manage any more...breathing heavily like I'd got pneumonia...it felt like I was going to have a heart-attack and when I came to the work-place I felt like I was going to throw up*.

Home and family were acknowledged and seen in a positive light:

*I was happy, I had a very stable family, both my own family, that's my husband and my children, but also my old family, my siblings and so on. They could acknowledge me – that of course I was a very precious and particularly competent person (laugh)*.

### Nagging darkness

These narratives also pointed to an "external" source, but the onset was "insidious". The informants only gradually became aware of their condition. Their recurrent depressive moods were seen as caused by nagging outside circumstances, such as cultural and social pressures or seasonal variations. Two of the men declared:

*The strange thing is that it always happens in springtime...when winter turns into spring. I guess it's something to do with the light*.

*I guess you could call it a fall depression...when it starts getting darker outside*.

For others it was long standing social pressures that clouded their spirits. One young woman had suffered bullying:

*I didn't know it was depression at first, but that I was bullied all through school, from the age of six until my first year in college*.

She described herself as a survivor, growing up in a family where both parents and siblings were chronically ill and without education or work. She herself managed to get a university degree, which helped her break the family pattern.

Two of the men talked about how family engagements and commitments contributed to their overstrain. One of them shouldered primary responsibility for his children and for his seriously ill wife:

*My children are everything to me...I had too many responsibilities and when I began to reflect on how much I did within a 24-hour period and how I managed it all...The fact is that it would've been too much for anyone*.

Although external circumstances were the source defining the category "nagging darkness", some of the informants also ascribed their illness to internal factors, such as hormones, genes or heredity.

I think it [depression] is due to a lack of hormones, that is, a consequence of being exposed to stress over such a long period of time. (woman)

An older man linked his recurrent depressions primarily to all too heavy responsibilities at work and at home. In addition he pondered about hereditary vulnerability, as his father and brothers also had depressive tendencies.

### Blackout

The "Blackout" narratives expressed a "sudden" onset of something that originated "internally", within the mind itself. This category consisted of few informants and only women. One of the women felt as if she was suicidal and about to lose her mind. She described the onset as an "unexplainable" acute anxiety attack at night. She could not breathe, panicked and rushed towards the balcony door:

*That I...that I...that I was kind of on my way out...on my way to open the door...to jump*.

She continued to talk about how she became afraid of herself and felt as if she was possessed by a dark force. Later in the interview she said:

*It's creepy not being able to steer your body...I thought I'd gone nuts*.

Shortly after, it was discovered that this woman suffered from heart failure. This could have contributed to her nightly anxiety and breathing difficulties. However, she herself considered her condition to be psychological.

Another woman talked about how she lost her foothold in life, felt almost unconscious, as if "in a black hole" from which she could not escape. Looking back she described herself as a zombie:

*When I looked at my photos from this period, there's a dead person there, not a living one*.

### Slowly suffocating

These illness narratives, also found only among women, described the course of depression as "insidious", something that gradually affected them negatively. The source was "internal", their personality or their way of handling life. They talked about themselves in disparaging terms, blaming themselves and using expressions such as "I'm strange", "always tired", "lazy" or "a failure". They described a negative spiral with no definite beginning or end, growing insidiously and gaining downward momentum. Statements included: "Even if I felt good, I would still feel worse than others" and "My personality is inclined to becoming depressed".

Although some of these women also described painful life events, they considered the source of their mood disorder to originate within their own personality. For instance, one young informant had experienced sexual abuse. Still, she blamed herself for being too melancholy and argued that in general "girls take things too seriously".

Many of the women regarded their shortcomings as intrinsic to their personality, choking their spirits and making them overly tired. They were burdened by feelings of guilt and shame, frequently related to never-ending female responsibilities. They thought they were never good enough:

*And then it's about doing one's share. You can't just stay at home and hang around and not...and leave the children at day care and stay home and...It was hard to know what to tell people...I withdrew instead and wondered even more about what others thought of me*.

The oldest woman in our study had been depressed since her teens without really understanding what it was about or how to get help. She drew back early in life, not allowing herself to go out and have fun with friends, postponing dates at the last minute:

*I thought I was different from my age-mates. I myself thought I was strange. And later I understood that in fact I was often very sad, because I withdrew, went aside, wanted to sit alone*.

She sought doctors with a wide variety of complaints, hoping they would be able to read between the lines and understand her inner moods. Finally at middle-age, she managed to get help from a general practitioner who referred her to psychotherapy. But in the end, she found it easier to seek primary health care, which had less of a stigma for her. Therapy helped her to verbalize her thoughts and feelings. She came to the conclusion that it was her low sense of self-esteem, as well as the high demands made on women traditionally (to keep the family functioning, the children clean, and the home spic and span) that had frightened away her lust for life:

*It's the woman who's been shamed when it doesn't function*.

She summed it all up in an existential longing:

*The bottom line I think...is some kind of thirst for love (sighing)*.

### Gendered trajectories

In this study, the informants' explanations for their depression were strongly gendered. The men were more likely than the women to describe themselves as having been struck by illness. They felt they were victims of external forces and that their bodies were heavily engaged. With few exceptions, the participating women described depression primarily as a lingering tiredness resulting from their personality. They blamed themselves and deemed themselves responsible for their depression.

## Discussion

Our analysis showed that the informants' understanding of their depression could be characterized by how they told their story; how the course and source were presented as sudden or insidious, external or internal, involving either the body or the mind. Informants described different trajectories into depression that could be represented by four symbolic illness narratives. These narratives showed gendered patterns. Men's stories most resembled the "Struck by lightning" narrative with sudden onset and dramatic physical symptoms. Their bodies were harmed by something external. Most of the women explained their depression as "Slowly suffocating", a pattern with insidious onset, self-accusations regarding their own shortcomings and personality failures, and an overriding sense of responsibility for their situation.

These gendered differences coincide with findings from our earlier study of media portrayals of depression [40]. However, our four narratives are by no means fixed entities with absolute boundaries. Rather the boundaries are porous [37] and we identified exceptions. A few of the women gave external explanations and some of the men considered their inherited personality as contributing to their depression. These "deviant cases" are of critical importance in that they may well embody the potential for changing gender patterns.

Kangas [16] found a similar pattern of externalizing and internalizing etiology in lay narratives of depression. Having an external view meant that the subject was not responsible for falling ill, whereas those with an internal view regarded depression as their own fault. However, Kangas performed no gender analysis. McMullen [41] showed that depressed women perceive depression and being "down" as the fault of the individual. Kilmartin's [21] description of masculinity as "acting out" and femininity as "acting in" likewise corresponds to the gendered patterns we identified. These patterns not only color the patient's understanding, but also impact on the doctor's perceptions.

We hold that the four types of narratives in our analysis can be recognized and utilized by the doctor in encounters with depressed patients. For instance, patients that are suddenly struck might benefit from exploring their home and inner situation; those heavily blaming themselves might gain a wider perspective by considering psychosocial circumstances. We suggest also that these depression narratives are expressions of gender constructions. To negotiate a successful mutual understanding in the doctor-patient relationship, gender awareness is crucial. Attentiveness to such constructions may help physicians recognize and diagnose depression, as well as prescribe treatment and aid recovery.

Our gendered narratives also suggest why women to a greater degree than men are diagnosed with depression. The characteristics of these narratives correspond in varying degree to the diagnostic criteria of DSM-IV [42], making it more difficult, for example, to recognize "Struck by lightning" as depression. Thus, making it more difficult to recognize men as depressed.

Kessler and colleagues ([43] have categorized three types of symptom attribution styles – psychologizing, normalizing and somatizing. These divergent patterns of symptom attribution correspond in large measure to how women and men in our study located the source of their depression on the inside or the outside of themselves. Kessler and colleagues ([43] found that physicians were likely to detect the psychologizers (most often women), who explain their problems in psychological terms; and to miss the normalizers (most often men), who have a hard time accepting any symptoms of disease. Somatizers were also likely to be detected.

However, Thomas-MacLean and colleagues [44] point out that physicians have to rule out all possible somatic illnesses before making the diagnosis depression, in that physical symptoms may mask emotional conditions. In our study many of the men might have been labelled somatizers, as they described the onset of depression in physical terms. As women emphasize psychological factors, somatic illnesses might go undetected. For instance, one of the informants with the "Black out" narrative proved later to have a heart failure that was nearly missed.

Labelling women as depressed is a modern phenomenon. Hirshbein [45] explored the emergence of depression as a diagnosis in American psychiatry from a historical viewpoint. She found that "depression" did not emerge as a specific disease entity with concrete criteria until new drugs were tested in the 1950s. These criteria were developed in hospitalized patient populations where two-thirds or more were women. By the 1980s, depression was described as the "common cold" of mental illness. During the process Hirshbein maintains, a number of assumptions – particularly concerning women – were buried within the "scientific" definition of the disease.

There is still no clear biomedical explanation as to why women are diagnosed with depression twice as often as men. Current medical textbooks often state this prevalence difference without reflecting more seriously about possible causes [46]. Psychiatric conditions may also be understood as symptomatic embodiments of cultural tensions and power imbalances in society, i.e., as a "crystallization of culture" [47]. Kangas [16] views narratives about depression as powerful characterizers of society. However, these perspectives may be difficult to incorporate in clinical work. Thomas-MacLean and Stoppard [30] suggest that physicians' frustration with depressed patients results from a disjunction between their biomedically oriented training and their ongoing exposure to their patients' lives and social realities. "Physicians' training would benefit from the integration of a multidisciplinary perspective on depression, which would better reflect physicians' experiences in routine practice situations" [30]. We emphasize the importance of taking gender aspects into account, as these also reflect the influence of a changing society and culture.

When treating depressed women and men, gender consciousness is required. Considering depressive symptoms as culturally dependent opens an array of possibilities to health care professionals. We may focus on gendered cognitive patterns in therapy, and make gendered societal attitudes visible, attitudes that are often left unquestioned. Women's tendency to self-blame and self-imposed responsibility, for instance, might be identified and unburdened [7]. Men, on the other hand, may need encouragement to express their emotions, establish close relationships, and accept responsibilities [21].

Focusing on men's accounts of depression, Emslie and colleagues [9] found that values associated with hegemonic masculinity frequently were incorporated in their narratives. However, they also identified alternative patterns of expression, such as being creative, understanding and compassionate. In our study, men emphasized work capacity and self-efficacy – "a real man can manage himself". This attitude makes it difficult to admit personal failure and to seek psychological help. Gender researchers have pointed out that conforming to the dominant norms of masculinity and femininity may well be damaging to health, while becoming aware of gendered patterns may lead toward more health-promoting strategies [9,13,21,24]. For men, this might be a question of acting in and for women of acting out.

Despite the higher rate of depression among women, Courtenay [48] maintains that women adopt healthier behavior and personal practices than men, whose signifiers of "true" masculinity are largely unhealthy. Dominating characteristics of masculinity – such as strength, courage, and being "one of the boys" – may encourage risk-taking and suicidal behavior. Educating men about masculinity may help them access their feelings and build on positive masculine qualities. This could be done in part by reframing masculine strategies, emphasizing the strength and courage needed to risk expressing feelings and to show vulnerability [9,21,26,48]. Further, it is important to legitimate men's engagement with health services and challenge preconceived normative notions that men will not, or cannot, talk and share experiences, particularly health-related experiences [13].

Encouraging gender consciousness in consultation may contribute to a rethinking and re-evaluation of self, relieving blame. Previous research on consultation with women patients emphasizes the importance of finding strategies to empower women by recognizing their salutary capacities [49,50].

### Limitations and strengths

Our data emanated from individuals who had personal experience of depression. A limitation might be that we recruited only those prepared to describe their experiences, and not those reluctant to relate their story [14] or constrained by a "maintaining face" attitude [17]. On the other hand, that our informants had a lot to tell may not have been a matter of recruitment, but rather of methodology: Malterud's key questions [34] guided them past their initial hesitation as to where to begin. Emslie and colleagues [9,15] found quite similarly that both the men and women were willing to talk about their experiences of depression when asked open-ended questions, which allowed them to focus on issues of importance to them. Thereto, our interviews were conducted off-hours, outside the consultation time schedule, allowing for both a freer disposition of time and of thought. Informants rarely mentioned biomedical parameters such as serotonin or hormones, but instead responded to the opportunity of telling their unique story – the course and source of their becoming depressed.

Perhaps more surprisingly, informants seldom mentioned childhood trauma. In a number of previous studies depressive negative thoughts have been found to emanate from childhood or teenage experience [9,16]. Our informants were generally vague about childhood. Only one woman, with a previous history of psychotherapy, identified her distress as beginning in her teens. The Swedish health care system is tax subsidized, universal and nationally programmed. Within primary care, consultation-therapy that sharply focuses childhood experience is clearly the exception; the overwhelmingly dominant emphasis is on the "here and now".

Although the sample is quite small, we believe that given its diversity it captures perceptions of depression in a contemporary Swedish context. However, recruitment of informants of ethnic minority background, as well as of more diverging ages, would undoubtedly have deepened and complicated the picture. A wider age range would open for an exploration of how depression is viewed, perceived, and explained across the life course, as well as how gender impacts on that course. Other issues in need of further research include the gendered pattern of help seeking, as well as patient response to receiving a diagnosis.

As the study was conducted in (northern) Sweden, we do not claim that results can be generalized to all societies. A special strength of this study is that it relies on lay informants outside an Anglo-American context, heretofore the dominant area of investigation. Nevertheless, many of the explanations and perceptions of depression found in our study correspond to those of earlier studies [9,16], a correspondence that supports the validity of our findings. Still, the need remains to explore narratives of depression outside the boundaries of modern Western society.

Another strength of this work, is the focus on *both *men's and women's perceptions and explanations of depression. A majority of earlier studies of lay narratives have focused on either women's or men's experiences. This study, in accordance with some earlier studies of men [9,11,13], underscores the possibility of recruiting men open and willing to share their narratives of depression. However, when comparing women and men, there is always a risk that instead of challenging gender norms findings tend to consolidate stereotypical portraits. We hope that by presenting a model that accounts for both perceived cause and source, that lays emphasis on exceptions and contradictions, and that derives its framework from social constructionism, we open for a more complex and comprehensive understanding of depression.

## Conclusion

Exploring and identifying gendered trajectories into depression through patients' own explanations may be helpful in interpreting, diagnosing, and treating men and women. Recognizing symbolic illness narratives and taking gender into account may work as a salutary tool in consultations. It may also help disclose the course and source of depression, bridge links between external and internal pressure, minimize self-blame, and at the same time encourage the development of new, healthier practices of how to be a man or a woman.

## Competing interests

The authors declare that they have no competing interests.

## Authors' contributions

The study as a whole was initially conceived by UD and EJ, and the two of them conducted all of the interviews. The focus for this article developed in a gender network group in which all five authors participated. Initial coding was performed by UD and EJ, all five authors took part in developing and refining the analysis. UD wrote the initial article draft; EJ and CB were active in the redrafting process; AL and AH contributed with critical suggestions and additions. All five authors approved the final manuscript.

## Pre-publication history

The pre-publication history for this paper can be accessed here:


